# Protocol: analytical methods for visualizing the indolic precursor network leading to auxin biosynthesis

**DOI:** 10.1186/s13007-021-00763-0

**Published:** 2021-06-22

**Authors:** Molly Tillmann, Qian Tang, Jerry D. Cohen

**Affiliations:** Department of Horticultural Science and Microbial and Plant Genomics Institute, University of Minnesota, St. Paul, MN USA

**Keywords:** Auxin biosynthesis, Stable isotope labeling, LC–MS, Metabolic inhibitors, Pathway analysis

## Abstract

**Background:**

The plant hormone auxin plays a central role in regulation of plant growth and response to environmental stimuli. Multiple pathways have been proposed for biosynthesis of indole-3-acetic acid (IAA), the primary auxin in a number of plant species. However, utilization of these different pathways under various environmental conditions and developmental time points remains largely unknown.

**Results:**

Monitoring incorporation of stable isotopes from labeled precursors into proposed intermediates provides a method to trace pathway utilization and characterize new biosynthetic routes to auxin. These techniques can be aided by addition of chemical inhibitors to target specific steps or entire pathways of auxin synthesis.

**Conclusions:**

Here we describe techniques for pathway analysis in *Arabidopsis thaliana* seedlings using multiple stable isotope-labeled precursors and chemical inhibitors coupled with highly sensitive liquid chromatography-mass spectrometry (LC–MS) methods. These methods should prove to be useful to researchers studying routes of IAA biosynthesis in vivo in a variety of plant tissues.

**Supplementary Information:**

The online version contains supplementary material available at 10.1186/s13007-021-00763-0.

## Background

Plant life is characterized by strictly regulated developmental events that achieve optimum growth and reproduction. This is accomplished through an extremely complex hormonal signaling network in which the plant growth hormone auxin plays a central and defining role. To this end, auxin helps regulate almost all aspects of plant growth and development including embryogenesis, tissue architecture and tropic responses [1]. Maintenance of auxin homeostasis involves multiple pathways for the biosynthesis of indole-3-acetic acid (IAA), the principal auxin in plants, and several regulatory pathways as well as subsequent catabolic events. These additional input/output processes include conjugation and hydrolysis of sugar and cyclitol conjugates, amino acid, peptide and protein conjugates, formation and β-oxidation of indole-3-butyric acid as well as deactivation by ring oxidation of IAA and its amino acid conjugates [2, 3]. Nevertheless, how much IAA is made and accumulates remains the critical regulatory event in many aspects of plant development [4].

**Fig. 1:**
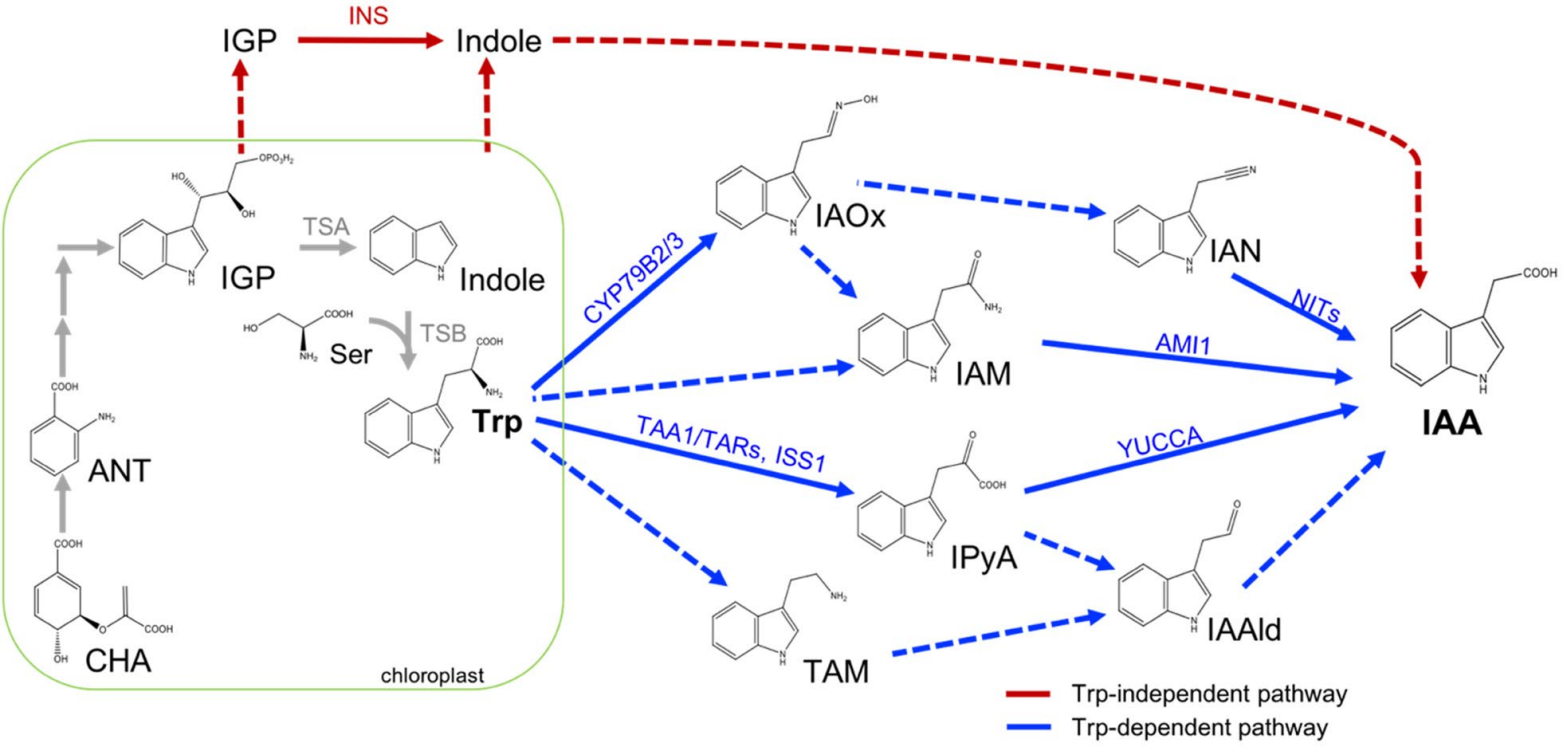
Major pathways for IAA biosynthesis. Solid arrows refer to pathways with enzymes identified in at least one species, and dashed arrows to undefined ones. AMI1, indole-3-acetamide hydrolase-1; ANT, anthranilate; CHA, chorismic acid; IAAld, indole-3-acetaldehyde; CYP79B2/3, cytochrome P450 (79B2/3); IAM, indole-3-acetamide; IAN, indole-3-acetonitrile; IAOx, indole-3-acetaldoxime; IGP, indole-3-glycerol phosphate; INS, indole synthase; IPyA, indole-3-pyruvic acid; ISS1, Indole Severe Sensitive 1; NIT, nitrilase; Ser, serine; TAA1, tryptophan aminotransferase of Arabidopsis 1; TAR, tryptophan aminotransferase-related; TAM, tryptamine; Trp, tryptophan; TSA, tryptophan synthase α; TSB, tryptophan synthase β; YUCCA, Arabidopsis flavin monooxygenase

**Fig. 2:**
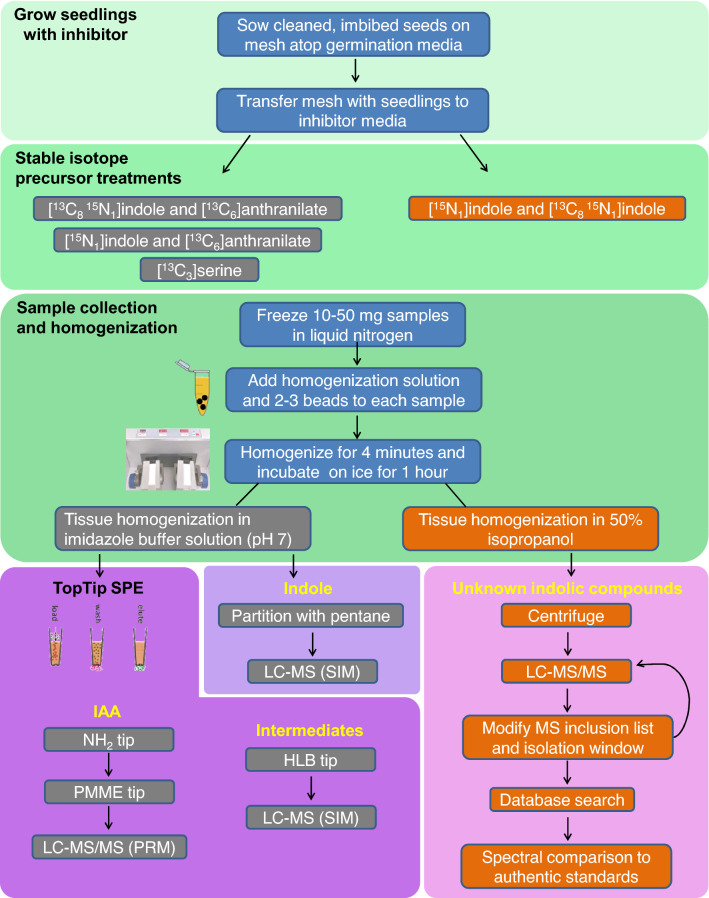
Workflow summary for labeling and analysis of the auxin metabolic network. Various labeling and analysis techniques are used to investigate different aspects of auxin biosynthesis. For absolute quantitation, internal standards are added to samples prior to homogenization

Although several biosynthetic pathways for the bioactive auxin IAA have been proposed, many of them have not been well defined and flux information is largely lacking (Fig. 1). The predominant biosynthetic route to IAA in *Arabidopsis thaliana* is widely believed to be through the YUCCA pathway, in which the amino acid tryptophan (Trp) is converted to indole-3-pyruvic acid (IPyA), which is then converted to IAA by YUCCA flavin monooxygenase enzymes [5]. Species-specific evidence for the synthesis of IAA from Trp through indole-3-acetaldoxime (IAOx), which is converted to indole-3-acetamide (IAM) and sometimes an indole-3-acetonitrile (IAN) intermediate has been shown in *Arabidopsis* [6, 7]. Other potential intermediates of IAA synthesis downstream of Trp have been proposed, such as indole-3-acetaldehyde (IAAld) [8–10] and tryptamine (TAM) [11], though their places within the web of auxin biosynthesis have not been well detailed. A Trp-independent route has also been proposed based on tryptophan synthase mutants, metabolic flux analysis and in vitro analyses, in which indole or another upstream compound serves as the IAA precursor [1, 12–14]; however, unbound chemical intermediates, if they are involved in this pathway, have as yet not been identified [15]. The purpose of this protocol is to describe improved techniques for characterization of the auxin metabolic network utilizing recently discovered chemical inhibitors and technical advances in mass spectrometry (Fig. 2). These tools will allow researchers to characterize auxin biosynthesis during specific developmental events or environmental responses.

Metabolic inhibitor approaches are complementary to genetic and biochemical studies and are particularly useful in studying IAA biosynthesis. While auxin biosynthesis mutants may have severe developmental defects that alter growth and confound comparisons to wild type plants [16], biosynthetic reactions can be turned off at specific developmental time points with chemical inhibitors. Additionally, genetic redundancy can be overcome by inhibiting an entire enzyme family with a single chemical treatment [17]. Such is the case with inhibitors targeting both steps in the YUCCA pathway. The YUCCA enzymes are encoded by multiple genes in *Arabidopsis thaliana* and mutations in small sets of these genes encoding the flavin monooxygenase proteins results in significant morphological defects [18]. A number of chemical inhibitors have been developed to inhibit the YUCCA pathway of auxin biosynthesis (Table 1), providing valuable tools to study the function of this pathway in different plant tissues and environmental conditions. Similarly, TAA1/TAR/ISS1/VAS1 (Tryptophan Aminotransferase of Arabidopsis 1/ Tryptophan Aminotransferase Related/ Indole Severe Sensitive 1 and reversal of sav3 phenotype 1) form a set of enzymes with overlapping biochemical functions that catalyze the penultimate step in the IPyA pathway [19]. Alternative aromatic amino acid substrates, such as L-kynurenine, can act as competitive inhibitors of tryptophan aminotransferase and a series of potent inhibitors have been developed to pyridoxal phosphate-dependent enzymes with enhanced specificity to TAA1 and related enzymes (“pyruvamines”; see Table 1) [20].

**Table 1:** Some chemical inhibitors of auxin biosynthesis

Inhibitor name	Representative structure(s)	Target	Mode of action	Reference
BBo	3-chlorophenylboronic acid, 4-biphenylboronic acid	YUCCA	Competitive inhibitor	[50]
PPBo	4-phenoxyphenyl-boronic acid	YUCCA	Competitive inhibitor	[50]
Ponalrestat	2-(3-(4-Bromo-2-fluorobenzyl)-4-oxo-3,4-dihydrophthalazin-1-yl)acetic acid	YUCCA	Substrate antagonist	[51]
Yucasin	5-(4-chlorophenyl)-4H-1,2,4-triazole-3-thione	YUCCA	Competitive inhibitor	[52]
Yucasin DF (YDF)	5-[2,6-difluorophenyl]-2,4-dihydro-[1,2,4]triazole-3-thione	YUCCA	Competitive inhibitor	[53]
Pyruvamines (PVM) “Type I compounds”	PVM1169; L-alpha-(aminooxy)-3-(naphthalen-2-yl)propanoic acid	TAA1	Competitive inhibitor	[20]
Pyruvamines (PVM) “Type II compounds” (Derivatives of Type I compounds)	PVM2153; Benzene propanoic acid, 3,4-dichloro-α-[(1,3-dihydro-1,3-dioxo- 2H-isoindol-2-yl)oxy]-, methyl ester	TAA1	Competitive inhibitor	[20]
L-Kynurenine (Kyn)	(2S)-2-Amino-4-(2-aminophenyl)-4-oxobutanoic acid	TAA1	Alternative substrate/ Competitive inhibitor	[54]
AVG	Aminoethoxyvinyl-glycine	TAA1	Slow-binding inhibition	[55]
AOPP	L-aminooxyphenyl-propionic acid	TAA1	Competitive inhibitor	[55]
AOA	Amino-oxyacetic acid	TAA1		[55]
AOIBA	2-amino-oxyisobutyric acid	TAA1		[55]
Indoleacrylic acid	trans-indole-3-acrylic acid	Trp synthase β and α	Allosteric inhibitor	[56, 57]
(1-Fluorovinyl)glycine	α-(1′-fluoro)vinyl glycine	Trp synthase β	PLP-enzyme mechanism-based inhibitor	[58]
Arylsulfide phosphonates	[4-[(2-aminophenyl) sulfanyl]butyl] phosphonic acid	Trp synthase α	Transition state analog	[59, 60]
Indoline-5-sulfonamides	1-(2-Fluorobenzoyl)-N-methyl-5-indoline sulfonamide N-Methyl-1-[(5-methyl-2-thienyl)carbonyl]-5-indolinesulfonamide	Trp synthase inter- subunit interface	Allosteric inhibitor	[61]
Sulfolane and indole-5-sulfonamide	GSK1, (3R,4R)-4-[4-(2-chlorophenyl)piperazin-1-yl]-1,1-dioxothiolan- 3-ol); GSK2, (1-[2-fluorobenzoyl]-N-methyl-2,- 3-dihydro-1H-indole-5-sulfonamide)	Trp synthase inter- subunit interface	Allosteric inhibitor	[62]
Aryl sulfonamides	[F9]; N-(4’-Trifluoromethoxy benzenesulfonyl)-2-aminoethyl Phosphate	Trp synthase β	α-Site allosteric ligand	[63]
Benzamide	N-(4-Carbamoyl benzyl)-5-(3-chloro phenyl)-1,2-oxazole-3-carboxamide	Trp synthase α	α-Site ligand	[64]

The issues of redundancy with tryptophan synthase (TS) are a bit different. *Arabidopsis* and maize have two copies of the genes that encode each of the two proteins that form the αββα heterodimeric complex that catalyzes the formation of tryptophan from indole-glycerol-phosphate and serine in the plastids. In addition, maize has genes BX1 and IGL for TSα-like cytosolic enzymes that serve as sources of free indole [21]. Arabidopsis also has a cytosolic TSα-like enzyme encoded by the indole synthase (INS) gene [22]. TS is, however, a well-researched and highly conserved bi-enzyme complex [23] such that inhibitors are available (Table 1) that target specifically TSα, TSβ as well as the 25-Å long tunnel to the β-subunit where indole diffuses in order to participate in the TSβ pyridoxal 5’-phosphate-mediated β-addition reaction with serine. Determining the possibility of a tryptophan-independent pathway is largely dependent on having Trp auxotroph mutants, which are difficult to obtain due to redundancy of Trp synthase genes and the fact that mutations in both copies of TSβ are seedling lethal [12, 13, 24]. The protocols described here largely overcome these issues by employing chemical inhibitors, and can complement genetic studies.

Mass spectrometry (MS) has historically been and continues to be an important technique in deciphering routes of auxin biosynthesis, enabling accurate quantitation of IAA and its precursors, identification of intermediates, and tracking of isotopic labels through distinct pathways. Quantitative methods for IAA and precursor analysis by MS have been invaluable tools in elucidating auxin biosynthesis pathways and have continuously evolved over time with advances in analytical sensitivity and resolution [4, 25–29]. Stable isotope tracing experiments also lend insight into auxin biosynthesis when plant tissue is supplied with one or more labeled precursors, such as indole and/or anthranilate [30–32], and label incorporation into suspected downstream intermediates is monitored to determine whether synthesis from the labeled precursor has occurred. This approach can also provide information regarding direction of flow and flux through different steps [6]. Additionally, labeled precursors that are unique to one pathway in particular can be applied to measure contributions of a specific pathway to the IAA pool [5, 33].

## Results and discussion

In this paper, we describe methods utilizing metabolic inhibitors coupled with a modified approach of isotope dilution/tracing and using liquid chromatography–high resolution-mass spectrometry (LC-HR-MS) for qualitative and quantitative analysis of a comprehensive set of IAA precursors and IAA itself to characterize auxin biosynthesis in *Arabidopsis* (see Additional file 1). A distinct advantage of this method is its ability to resolve potential precursor compounds by chromatographic retention, absolute mass and by elemental composition, enabling complex mixtures of different stable isotopes (for example, multiple labeled compounds with ^13^C and ^15^ N can be resolved) to be used in the experimental procedures (see Additional file 2). Readers may also consult a complementary paper that was published while this manuscript was in preparation [34]. Growing seedlings on fully ^15^ N-labeled media as described here enables accurate quantitation of biosynthetic intermediates by reverse isotope dilution, using unlabeled internal standards which are typically more readily available than isotopically labeled standards [35]. The addition of one or more ^15^ N atoms at a mass addition of 0.9970 can be resolved from the more abundant natural occurrence of ^13^C, which is 0.0034 heavier than ^12^C, which improves the utility of this approach when using high resolution analysis. Seedlings are first germinated on nylon mesh and are easily transferred onto media containing chemical treatments at the desired developmental time point. Next, stable isotope-labeled precursor compounds are fed to the plant. Labeled serine is used as a tracer for Trp-dependent biosynthesis specifically [33], while labeled indole and anthranilate can feed into both Trp-dependent and Trp-independent pathways [19, 31, 32] (Fig. 1). The techniques described here offer several advantages over previously described methods in their ease of preparation, high level of sensitivity, capacity for monitoring many compounds at once (see Additional file 2), and the ability of high resolution analysis to distinguish between different ‘heavy’ atoms, as might be required with [^13^C_1_]IAA and [^15^N_1_]IAA labeling products. As shown in Additional file 1, the use of multiple labels makes it easy to see that the addition of the tryptophan monooxygenase inhibitor YDF increases the incorporation of labeled indole into IAA but decreases labeling from labeled anthranilate and to a lesser degree from labeled tryptophan. Furthermore, this IAA labeling pattern for labeled indole and anthranilate is not reflected in any of the proposed intermediates following YDF treatment.

We also describe a technique for identifying novel intermediates based on the characteristic quinolinium ion produced from MS fragmentation of 3-substituted indolic compounds. This method involves using a series of injections of the same sample with increasingly narrow mass ranges, similar to the methods utilized by Yu et al. [36] and Tang et al*.* [37] where they targeted and identified novel indolic compounds. By monitoring exact masses of [^13^C_8_, ^15^N_1_]- and [^15^N_1_]quinolinium ions after treatment with [^13^C_8_, ^15^N_1_]- and [^15^N_1_]indole, this method can identify unknown compounds synthesized downstream from indole. A similar approach would likely be applicable in investigations of other classes of compounds that form characteristic signature ions. High resolution accurate mass analysis significantly reduces factors such as false negative molecular ions, low abundance ions, multiple isomers, and matrix effects, which otherwise would make it difficult to confirm possible compound identities.

## Materials and methods

### Materials

#### Growing, labeling, and collecting plant material

Wild-type Columbia-0 ecotype *Arabidopsis thaliana* seeds or specific metabolic mutant lines need to be surface sterilized sodium hypochlorite then imbibed for 5–10 days at 4 °C to promote uniform germination. Typically seeds would be sown in a single row onto 20 μm nylon mesh covering the agar growth medium.

- 20 μm nylon mesh (Sefar, 03–20/14), cut into 9 cm $\times$ 9 cm squares and autoclave sterilized with 45 min sterilization time at 121 °C
- Sterile deionized water
- Forceps, flame sterilized
- 10 cm $\times$ 10 cm square Petri dishes (Fisherbrand, FB0875711A)
- Dilute bleach solution for seed sterilization: 20 mL concentrated regular liquid bleach (Clorox), 80 mL deionized water, 20 μL Tween 80 (Sigma-Aldrich, P1754)
- Plant growth medium: *Arabidopsis thaliana* salts (ATS) [38]
- KimWipes delicate task wipes (Kimberly-Clark, KC34155EXL)
- 1.5 mL microcentrifuge tubes (Fisherbrand, 05–408-129)
- Liquid nitrogen
- Dry ice
- One or more isotopically labeled precursor solutions in aqueous ATS salts (see Notes 1 and 2; see Table 2 for description of example labeling strategies):
  - 3 mM [^13^C_3_]L-serine (Cambridge Isotope Laboratories, CLM-1574-H)
  - 500 μM [^13^C_6_]anthranilate (Sigma-Aldrich,709,530)
  - 500 μM [^15^N_1_]indole (Cambridge Isotope Laboratories, NLM-792)
  - 500 μM [^13^C_8_, ^15^N_1_]indole (Cambridge Isotope Laboratories, CNLM-4786–0)

**Table 2:** Labeling precursors used for different applications. Example labeling strategies employing different stable isotope-labeled precursors for studying IAA biosynthesis. These strategies can be used in combination with various inhibitors (Table 1) for targeted analysis of specific routes of IAA biosynthesis

Labeled precursor treatment	Germination Media	Purpose/Description
3 mM [^13^C_3_]Serine	[^14^ N] ATS	Traces synthesis of Trp and Trp-dependent pathway intermediates. [^13^C_3_]Serine is condensed with indole to give [^13^C_3_]Trp ([^13^C_3_]-label is incorporated into Trp sidechain)
500 μM [^13^C_6_]anthranilate and 500 μM [^15^N_1_]indole	[^14^ N] ATS	Multiple auxin precursors upstream of Trp are applied to monitor label incorporation into various intermediates through multiple pathways
500 μM [^13^C_8_^15^N_1_]indole and 500 μM [^15^N_1_]indole	[^14^ N] ATS	Multiple labeled forms of indole are applied to label indole-derived metabolites and potential IAA biosynthesis intermediates. LC–MS/MS analysis workflow for identifying candidate compounds is described in the Materials LC–MS analysis section
500 μM [^13^C_6_]anthranilate and 500 μM [^13^C_8_^15^N_1_]indole	[^15^ N] ATS	Growing seedlings on [^15^ N] ATS media enables rapid [^15^ N]-labeling of newly synthesized IAA and biosynthesis intermediates during early seedling development (Fig. 3) Unlabeled internal standards may be used for quantitation of IAA and biosynthetic intermediates in plant tissue grown on [^15^ N] ATS (reverse isotope dilution quantitation)

**Fig. 3:**
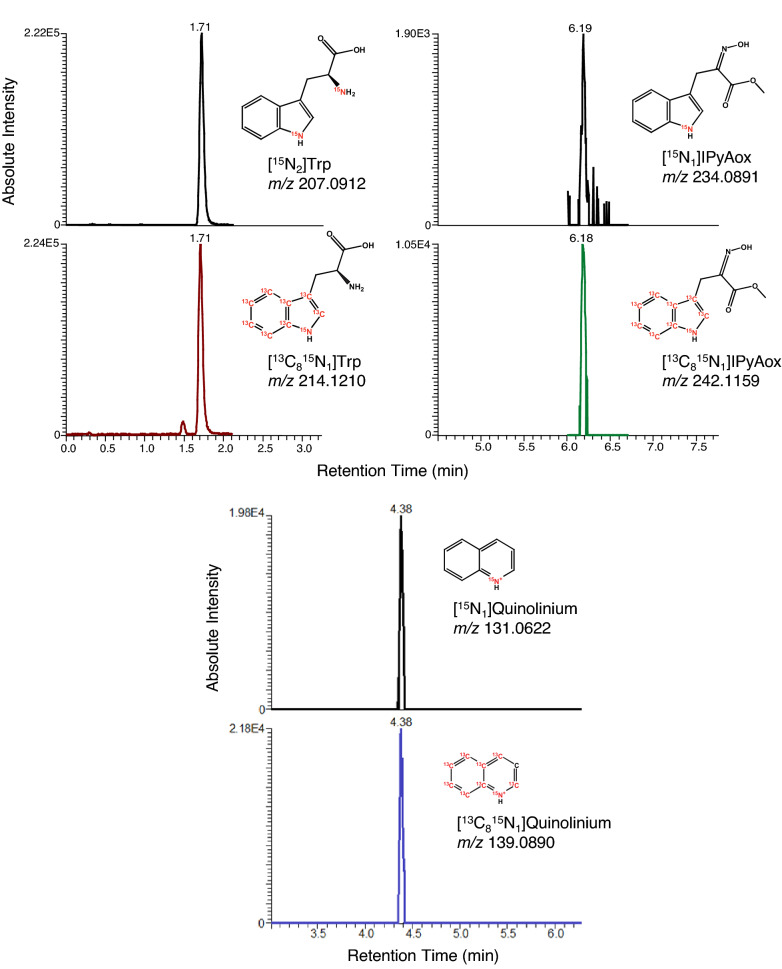
Representative results from analysis of Trp, IPyA, and IAA extracted from Arabidopsis seedlings. 13-day-old seedlings grown on ^15^ N media were subjected to mock inhibitor treatment (DMSO + ACN) for 22 h, then labeled with 500 μM [^13^C_8_, ^15^N_1_] indole for 1 h. [^15^N_2_]Trp, [^13^C_8_, ^15^N_1_]Trp, [^15^N_1_]IPyAox, and [^13^C_8_, ^15^N_1_]IPyAox were monitored in SIM mode. [^15^N_1_] IAA [^15^N_1_]quinolinium and [^13^C_8_, ^15^N_1_]IAA [^13^C_8_, ^15^N_1_]quinolinium transitions were monitored in PRM mode

#### Homogenization and extraction

Plant tissue samples, typically 20–50 mg, are homogenized for 4 min using a bead mill with three stainless steel balls and 40–100 μL of the extraction buffer/solvent.

- Tissue homogenizer (Retsch MM300)
- Microcentrifuge, temperature controlled at 4 °C
- Repeater pipette (Eppendorf M4)
- Homogenization buffer: 65% isopropanol, 35% 0.2 M imidazole (pH 7.0), 100 mM methoxyamine hydrochloride (CH_3_ONH_2_∙HCl) (Sigma-Aldrich, 226,904) for targeted analysis of IAA and biosynthesis intermediates (see Note 3); 50% isopropanol for analysis of unknown indolic compounds
  - For absolute quantitation, stable isotope internal standard is added into homogenization buffer. The amount of internal standard added to each sample should be similar to the amount of endogenous compound in the plant tissue (see Notes 4 and 5)

- Stainless steel beads for homogenization (1.6 mm diameter, Next Advance, SSB16)
- 2 mL screwcap tubes (Fisherbrand, 02–681-343)
- 10–200 μL Empty TopTips (Glygen, TT2EMT) and adaptors provided by Glygen for centrifugation
- Vacuum concentrator (SpeedVac, Savant)
- Additional materials required for specified extraction techniques are described in the sections below:

#### IAA extraction

Homogenized samples are incubated on ice for 50 min to allow isotopic standard equilibration with the endogenous IAA. They are then diluted tenfold with water such that ion exchange will be effective, centrifuged to remove solid materials, and loaded onto two consecutive SPE micro spin column (TopTips) steps, first ion exchange on an amino phase and then on an epoxide support.

- Bondesil-NH_2_ resin (Agilent, 12,213,020) suspended in water, 1:4 w:v
- IAA extraction solvents and solutions as described by Liu et al. [26]: hexane, acetonitrile, ethyl acetate, methanol, 0.2 M imidazole (pH 7.0), distilled water, 0.25% phosphoric acid (PA), 0.1 M succinic acid (SA, pH 6.0), 5:1 PA:SA solution
- Macro-prep epoxide support resin (Bio-Rad, 156–0000), suspended in 0.1 M sodium bicarbonate (pH 7.0), 1:4 w:v

#### Extraction of proposed IAA biosynthesis intermediates: Anthranilate, Ser, IAAld, IPyA, IAOx, IAN, IAM, indole

Homogenized samples are incubated on ice for 50 minutes to allow isotopic standard equilibration with the endogenous compounds, diluted 10-fold with water to allow proper interaction with the solid phase, centrifuged to remove solids, and loaded onto a SPE micro spin column (TopTips) containing hydrophilic-lipophilic balanced (HLB) resin conditioned with acetonitrile followed by 20% acetonitrile in water. After loading, the spin columns are washed with 5% acetonitrile and compounds are eluted with 80% acetonitrile.

- RENSA HLB resin (MIP Technologies, 92,001–0010) suspended in methanol, 1:5 w:v
- Acetonitrile: 100%, 80%, 20%, and 5% prepared in distilled water

#### Indole extraction

Indole is a very lipophilic and somewhat volatile compound that cannot be purified using the techniques used for the other compounds. Thus, its purification involves a simple solvent partitioning. It was important to select an apolar solvent with a boiling point below the melting point of indole. We found pentane to be well-suited as its boiling point is 36.0 °C, well below the indole melting point of 52.5 °C.

- Pentane

#### LC–MS analysis

UPLC utilizes a column with an end-capped octadecylsilane fully porous 1.8 µm silica resin with high carbon loading (20%) in order to obtain highest sensitivity for indolic compounds (see Additional file 2).

- Amber autosampler vials (ChromTech, 404,810) with 50 μL glass inserts (ChromTech, CTI-2405)
- Liquid chromatograph/mass spectrometer system: Dionex UltiMate 3000 UHPLC, Q Exactive mass spectrometer, Xcalibur software (Thermo Scientific)
- C_18_ HPLC column, 50 $\times$ 2.1 mm (Force, 9,634,252, Restek) with 0.2 μm precolumn filter (UltraShield, 25,809, Restek)
- Mobile phase: A, 0.1% formic acid in water; B, 0.1% formic acid in acetonitrile. Different LC–MS methods are used to target compounds of interest:
  - IAA analysis: Mobile phase gradient of 5% B (-1–0 min), 5–20% B (0–3 min), 20–80% B (3–6 min), 80% B (6–6.5 min) at a flow rate of 0.4 mL·min^−1^. Mass spectra are collected in positive ion mode in a parallel reaction monitoring (PRM) scan and the inclusion list contains ions of 176, 177, 178, and 182 m*/z*. PRM resolution is 17500 full width at half maximum (FWHM), automatic gain control (AGC) target is 2 × 10^5^, maximum ionization time is 50 ms (ms), isolation window is 2.0 m*/z*, and normalized collision energy (NCE) is 20. Ion source conditions are: spray voltage: 4.00 kV, capillary temperature: 275 °C, probe heater temperature: 300 °C, sheath gas: 30 arbitrary units, aux gas: 20 arbitrary units, S-lens RF level: 50.
    For analysis of all the listed intermediates except indole, inject 5–10 μL plant extract into the LC system with the following mobile phase gradient: 5% B (− 2–1 min), 5–15% B (1–3 min), 15–30% B (3–3.5 min), 30% B (3.5–5 min), 30–39% B (5–7.5 min), 39–80% B (7.5–8 min), 80% B (8–8.5 min) at a flow rate of 0.4 mL·min^−1^. Mass spectra are collected in selected ion monitoring (SIM) mode. SIM resolution is 70,000 FWHM with maximum ionization time of 200 ms and AGC of 5 × 10^5^. Ion source conditions are: spray voltage: 4.00 kV, capillary temperature: 275 °C, probe heater temperature: 300 °C, sheath gas: 30 arbitrary units, aux gas: 20 arbitrary units, S-lens RF level: 50. MS is set to acquire several segments of full scans each targeting 1–3 compounds. The segments are: 200–217 m*/z* (0–2.1 min), 157–173 m*/z* (2.1–3 min), 133–150 m*/z* (3–3.74 min), 170–188 m*/z* (3.74–5.4 min), 152–170 m*/z* (5.4–6 min), 227–245 m*/z* (6–6.7 min), 184–201 m*/z* (6.7–8.5 min).
    For indole analysis, 5–10 μL plant extract is injected with the following LC mobile phase gradient: 5% B (− 1–1 min), 5–30% B (1–3 min), 30–39% B (3–5.5 min), 39–80% B (5.5–6.5 min), 80% B (6.5–7 min) at a flow rate of 0.4 mL·min^−1^. Mass spectra are collected in SIM mode using a mass range of 110–132 m*/z*. SIM resolution is 70,000 FWHM with maximum ionization time of 200 ms and AGC of 5 × 10^5^. Ion source conditions are: spray voltage: 4.00 kV, capillary temperature: 275 °C, probe heater temperature: 300 °C, sheath gas: 30 arbitrary units, aux gas: 20 arbitrary units, S-lens RF level: 50.
    For analysis of compounds labeled by treatment with [^15^N_1_]- and [^13^C_8_, ^15^N_1_]indole, multiple injections of the same sample are made using a series of methods (A-D, described below) with different MS parameters. The same mobile phase gradient is used with each method: 5% B (− 2–2 min), 5–50% B (2–8 min), 50–85% B (8–10 min), 80% B (10–12 min) at a flow rate of 0.4 mL·min^−1^.
    - Method A: Scan groups consist of one full MS-SIM scan followed by four PRM scans. For the SIM scan, Orbitrap resolution is 70,000 full width at half maximum (FWHM) with maximum ionization time of 200 ms, automatic gain control (AGC) target of 5 × 10^5^, and scan range of 100–400 m*/z*. For the PRM scans, FWHM resolution is set to 17,500, maximum ionization time is 100 ms, AGC target is 2 × 10^5^, normalized collision energy (NCE) is 35, and isolation window is 20 m*/z.* Four variations of this method (used in four separate injections) with different values in the inclusion list are used to cover a range of *m/z* values for potential compounds of interest:
      - Method A.1: The inclusion list contains *m/z* values beginning at 130 and increasing by increments of 20 to 290 (130, 150, 170,… 250, 270, 290 m*/z*)
      - A.2: *m/z* values begin at 140 and increase by increments of 20 to 300
      - A.3: *m/z* values begin at 300 and increase by increments of 20 to 460
      - A.4: *m/z* values begin at 310 and increase by increments of 20 to 470
- Method B: LC–MS parameters are nearly identical to Method A, except that the isolation window is changed to 2 m*/z* and the inclusion list is designed to cover a 20 m*/z* range with values increasing by increments of 2 m*/z*. The inclusion list is customized to target features of interest observed using Method A. For example, to investigate a candidate peak identified in the 210 m*/z* scan filter from Method A, the inclusion list for Method B would contain 200, 202, 204, … 216, 218, 220 m*/z.*
- Method C: Again, LC–MS parameters are nearly identical to Methods A and B, except that the isolation window is further narrowed to 1 m*/z* and the inclusion list is customized to isolate the isotopomers of interest observed with Method B. Values within 1–2 m*/z* of the scan range containing features of interest found from Method B are included. For example, to target a peak observed in the 206 m*/z* scan filter from Method B, 205, 206, and 207 m*/z* should be added to the inclusion list of Method C.
- Method D: Scan groups include one full MS- SIM scan followed by one PRM scan. MS parameters are the same as those described above except that the NCE is 15 and the isolation window is ≤ 1 m*/z*. The inclusion list is customized to contain only the [^13^C_8_, ^15^N_1_]-, [^15^N_1_]-, and unlabeled molecular ions of interest with as much specificity as possible.

### Methods

#### Growing seedlings with inhibitor and stable isotope precursor treatments

Seedlings are grown in vitro on mesh squares, allowing them to be easily transferred to chemical inhibitor treatments at the desired timepoints. A liquid solution containing stable isotope-labeled precursors is then supplied to seedlings, and synthesis of isotopically labeled IAA and intermediates can be identified and distinguished by LC-HR-MS.

1. In a laminar flow hood, moisten sterile nylon mesh squares with sterile water and use forceps to place squares flat on germination media (see Note 7 and Table 2) in square Petri dishes.
2. Clean *Arabidopsis* seeds by shaking in 20% bleach solution for 5 min and rinsing 4 times with sterile water.
3. Sow seeds approximately 0.5 cm apart in single row on mesh.
4. Store plates at 4̊ C in the dark for 3–7 days to stratify seeds. Remove plates from cold and place vertically in growth conditions.
5. Transfer seedlings onto inhibitor media (Table 1) to begin auxin biosynthesis inhibition treatment (see Note 8). In a laminar flow hood, use forceps to gently lift mesh with seedlings from germination plates and lay flat onto plates containing inhibitor media. Cover plates and place vertically under growth conditions.
6. Begin isotopic labeling treatments by flooding plates with 3 mL of labeling solution (Table 2). Gently rock plate back and forth 5–10 times to ensure labeling solution covers entire mesh square. Cover plates and place flat under growth conditions for 0–24 h (see Note 8).
7. Collect samples by gathering 10–50 mg of plant tissue (see Notes 9 and 10), gently blotting away moisture on a KimWipe, and placing in a microcentrifuge tube. Immediately submerge tube in liquid nitrogen to flash freeze and place on dry ice. Store samples at -80̊ C until extraction.
8. Holding frozen samples on dry ice, add 20 μL of homogenization solution per 10 mg tissue and 2–3 beads to each sample.
9. Homogenize samples in tissue homogenizer for 4 min at 1500 RPM and incubate samples on ice for 1 h (see Note 11)
10. Add 90 μL of water to each homogenized sample per 10 μL homogenization buffer and shake tube to mix.
11. Centrifuge samples at 25,000*g* for 10 min. at 4 °C.
12. Prepare samples for LC–MS analysis using purification methods specified for compounds of interest:

#### Indole

Indole is a biosynthetic precursor of IAA and Trp, and is extracted by solvent partitioning.

13. Transfer 200 μL supernatant into a new tube. Add 100 μL pentane and vortex at room temperature for 5 min.
14. Spin samples a short time to clearly separate the organic and aqueous phases. Transfer the upper organic layer into a new tube. Save the aqueous phase for extraction of IAA (steps 16–21) or auxin biosynthesis intermediates (steps 22–26).
15. Evaporate pentane with vacuum concentrator and resuspend sample in 20 μL acetonitrile

#### IAA

IAA is typically present at low levels in plant tissues and can be extracted separately from other compounds using more selective methods for optimal detection. IAA samples are purified by solid phase extraction (SPE) using an amino (NH_2_) resin followed by a second step with polymethylmethacrylate epoxide (PMME) resin.

16. Prepare TopTips with NH_2_ resin for SPE according to Liu et al. [26]. Add 20 μL resin suspension per TopTip; wash with 50 μL each: hexane, acetonitrile, ethyl acetate; condition with 50 μL 0.2 M imidazole followed by 2 $\times$ 100 μL water
17. Load supernatant from step 11 onto prepared TopTips. For larger samples, 250 μL of supernatant can be loaded at a time and spun through. Reload and spin until all supernatant has been loaded. Wash with 50 μL methanol.
18. Exchange tubes under TopTip adapters to fresh 2 mL tubes for elution. Elute with 3 $\times$ 50 μL of PA, then add 25 μL of SA to each sample.
19. Prepare TopTips with PMME resin for solid phase extraction (SPE) according to Liu et al. (2012). Add 75 μL PMME resin suspension per TopTip; wash with 2 $\times$ 100 μL methanol; condition with 2 $\times$ 100 μL PA:SA.
20. Load samples onto prepared TopTips. Wash with 2 $\times$ 50 μL PA:SA.
21. Exchange tubes under TopTip adapters to clean 1.5 mL tubes for elution. Elute with 2 $\times$ 50 μL methanol. Reduce volume of each sample to approximately 20 μL with vacuum concentrator.

#### Proposed IAA biosynthesis pathway intermediates: Anthranilate, Ser, IPyA, IAAld, IAOx, IAN, IAM

Samples are prepared for analysis of biosynthesis intermediates by SPE using an HLB resin. SPE is an effective sample preparation technique for these compounds because it provides a high level of recovery and is relatively easy to use with large sample sets. IAA can also be extracted using the following method, but with some loss of sensitivity compared to methods described in the previous section.

22. Prepare TopTips with HLB resin for SPE. Add 25 μL resin suspension per TopTip; equilibrate with 2 $\times$ 50 μL 100% acetonitrile and 2 $\times$ 50 μL 20% acetonitrile.
23. Load supernatant onto prepared TopTips.
24. If highly sensitive detection and quantification of IAA is required, save 200–300 μL of supernatant for separate IAA analysis (steps 16–21). IAA may be extracted and analyzed simultaneously with precursor compounds if samples contain sufficiently high IAA levels (typically ≥ 2 ng).
25. Wash with 50 μL 5% acetonitrile.
26. Exchange tubes under TopTip adapters to clean 1.5 mL tubes for elution. Elute with 2 $\times$ 50 μL 80% acetonitrile. Reduce volume of each sample to approximately 20 μL using vacuum concentrator (about 10–12 min).

#### Unknown indolic compounds (double indole labeling samples)

An unbiased extraction method is used for discovery of unknown compounds synthesized from indole.

27. Transfer supernatant to a clean tube and centrifuge again at 25,000 g for 10 min. at 4 °C to remove all debris.

#### LC–MS analysis

Samples are analyzed using LC-HRAM-MS to chromatographically separate components of chemical matrix and obtain high resolution *m/z* data. Specific LC–MS methods are tailored for different sample types and analysis objectives (see method details in "Materials" section).

28. Carefully transfer each sample to a 50 μL glass insert so that no air pockets remain at the bottom of the insert. Assemble insert into autosampler vial with cap.
29. Inject 5–10 μL of sample for LC–MS analysis using methods described in the LC–MS analysis subsection of the Materials section.

#### Data analysis

##### IAA analysis

Extracted ion chromatograms (EICs) of labeled and unlabeled quinolinium ions generated by fragmentation of labeled internal standard and unlabeled endogenous IAA are viewed (see Additional file 2). Narrow mass ranges are used to filter out background noise.

30. Under the “Ranges” tab in “Chromatogram Ranges” in Xcalibur, set the chromatogram viewing options to display two mass ranges: 130.0641–130.0661 (corresponding to unlabeled quinolinium ion), and 136.0843–136.0863 ([^13^C_6_] quinolinium produced from [^13^C_6_]IAA internal standard). Under “Display” tab, check “Peak Area.” Use “peak selection” tool to select and calculate area of peaks corresponding to unlabeled IAA and the internal standard. Endogenous IAA levels can be calculated using isotope dilution [25, 39].

##### Targeted IAA precursor analysis

Peak areas from EICs of multiple compounds are determined using a script. Mass ranges surrounding the exact masses of ions produced from the compounds of interest, as well as their labeled forms synthesized from the supplied labeled precursors, are kept within a narrow window to exclude background noise.

31. Raw data files are converted to mzXML format using the msconvert tool from the ProteoWizzard software [40] prior to input into R. Quantitative data for each indolic compound is extracted using the Metabolite-Turnover script developed in the Hegeman lab (https://github.com/HegemanLab/Metabolite-Turnover, [41]). In this script, the ProteinTurnover [42] and the XCMS package [43] are employed to extract EICs for each isotopmer of IAA and intermediates. This quantification approach using linear regression [44] is preferred over that using peak area [39] when the MS data has high background noise due to low analyte abundance.
32. Exact masses for isotopomers of interest are calculated using the University of Wisconsin—Madison Biological Magnetic Resonance Data Bank exact mass calculator (http://www.bmrb.wisc.edu/metabolomics/mol_mass.php). Isotopomers of proposed IAA biosynthetic intermediates derived from several isotopic labeling strategies are listed in Table 3. (See Note 12)
33. In the data output csv files, the slope of each linear regression line represents the ratio of the respective isotopic trace to its monoisotopmer. This ratio is used to calculate the relative abundance of labeled compounds, allowing us to track label incorporation from upstream precursors into IAA intermediates through multiple pathways.

**Table 3:** The *m/z* values of isotopomers measured in the IAA and intermediates analyses

	M(+H)	^15^N_1_	^13^C_1_	^15^N_2_	^13^C_1_^15^N_1_	^13^C_1_^15^N_2_
ANT	138.0550	139.0520	139.0583		140.0553	
IND	118.0651	119.0622	119.0685		120.0655	
Trp	205.0972	206.0942	206.1005	207.0912		
Ser	106.0499	107.0469	107.0532			
TAM	161.1073	162.1044	162.1107	163.1014	163.1077	164.1047
IAOx	175.0866	176.0836	176.0899	177.0807	177.0870	178.0840
IAM	175.0866	176.0836	176.0899	177.0807	177.0870	178.0840
IAN	157.0760	158.0731	158.0794	159.0701		160.0734
IPyAox	233.0921	234.0891	234.0954		235.0925	
IAAldox	189.1022	190.0993	190.1056		191.1026	
IAA	176.0706	177.0676	177.0740		178.0710	
Quinolinium	130.0651	131.0622	131.0685			

	^**2**^**H**_**4**_	^**13**^**C**_**6**_	^**13**^**C**_**6**_^**15**^**N**_**1**_	^**13**^**C**_**7**_	^**13**^**C**_**7**_^**15**^**N**_**1**_	^**13**^**C**_**8**_^**15**^**N**_**1**_
ANT		144.0751	145.0721	145.0784		147.0788
IND		124.0853	125.0823	125.0886		127.0890
Trp		211.1173	212.1143		213.1177	214.1210
Ser						
TAM			168.1245		169.1278	170.1312
IAOx			182.1038		183.1071	184.1105
IAM			182.1038		183.1071	184.1105
IAN			164.0932		165.0965	166.0999
IPyAox		239.1122	240.1092	240.1156		242.1159
IAAldox		195.1224	196.1194	196.1257		198.1261
IAA	180.0957	182.0907	183.0878	183.0941		185.0945
Quinolinium	134.0902	136.0853				139.0890

##### Double indole labeling data analysis

Supplying plants two differentially labeled form of indole provides a way to identify indole-derived compounds, as downstream intermediates will incorporate both labels. These samples are analyzed in a series of LC-MS/MS injections, initially scanning broadly for formation of labeled quinolinium ions, and then narrowing in on precise ions in subsequent injections until a molecular ion can be identified and fragmented to provide further structural information.

34. Use LC–MS method A1 (described in Materials section “LC–MS analysis”) to identify potential features producing [^15^N_1_]- and [^13^C_8_, ^15^N_1_]quinolinium ions.
  - Under the “Ranges” tab in “Chromatogram Ranges” in Xcalibur, set the chromatogram viewing options to display three mass ranges: 130.0641–130.0661 (corresponding to unlabeled quinolinium ion), 131.0612–131.0632 ([^15^N_1_]quinolinium), and 139.0880–139.0900 ([^13^C_8_, ^15^N_1_]quinolinium).
  - Set “Scan Filter” to display chromatogram from the first ion of the inclusion list. Note any coeluting peaks present in both the [^15^N_1_]quinolinium and [^13^C_8_, ^15^N_1_]quinolinium mass ranges. Ions producing these peaks may have incorporated label from the indole treatments.
  - View chromatograms through each Scan Filter, keeping the same mass ranges specified above, and continue to note any candidate peaks with matching retention times.
35. Repeat workflow described in step 33 with method A2 to identify additional candidate features.
36. Perform subsequent injection with method B, aiming to narrow *m/z* windows containing the parent ions. View data using the same settings in Xcalibur that were used in step 33, taking note of retention times and scan windows that show the presence of coeluting peaks for both quinolinium labeled mass ranges.
37. Perform subsequent injection with method C to progress toward pinpointing molecular ions. This method should be tailored to candidate peaks that were identified in steps 33–35.
  - Using the same mass ranges settings to target quinolinium ion isotopomers, view EIC through each scan filter to identify the filter range containing the strongest quinolinium signal. The molecular ion is expected to be near (within 1 m*/z*) this value.
38. Customize method D to include approximate molecular ion values identified in step 35 in the inclusion list. Run sample using this method to obtain exact mass spectra.
39. Compare spectra produced from each of the three ions in the inclusion list of method D, taking note of exact mass differences between the major ions of different spectra. Exact mass differences of 0.9970, 9.0239, and 8.0268 m*/z* correspond to the differences between ^15^N_1_ and unlabeled, ^13^C_8_^15^N_1_ and unlabeled, and ^13^C_8_^15^N_1_ and ^15^N_1_, respectively.
40. If no molecular ions are observed, NCE settings in method D can be set to a lower intensity to preserve a greater abundance of molecular ion.
41. Unlabeled molecular ion and spectral data can be searched against mass spectral databases to identify potential compound identities: MassBank, METLIN, NIST Tandem Mass Spectral Library, m/zCloud, MS-DIAL.
42. Confirm compound identities by comparing retention time and mass spectral data against authentic standards.

#### Notes

1. 500 μM [^13^C_11_, ^15^N_2_]Trp (or other labeled forms of Trp) may also be used as a labeled precursor treatment [5, 12, 14, 31, 45]; however, results should be examined cautiously as high levels of exogenous Trp will feedback inhibit anthranilate synthase and anthranilate phosphoribosyltransferase [46–48], which may confound results.
2. To improve solubility, labeled indole can first be dissolved in a small volume of acetonitrile; anthranilate can first be dissolved in a small volume of isopropanol. Labeled indole and anthranilate concentrations were based on observation that significant labeling of IAA and biosynthetic precursors was achieved after incubation with 500 μM labeled precursor for 16 h, and labeled Ser concentrations were chosen to approximate endogenous Ser levels [49]. However, concentrations may be adjusted as needed for use in other systems (see Table 4).
3. IPyA and IAAld degrade quickly and need to be derivatized with CH_3_ONH_2_ to generate their oximes (IPyA-MeOx and IAAld-MeOx). Standards should be derivatized and freshly prepared.
4. For IAA quantitation by isotope dilution, mix 10 ng of stable isotope labeled-IAA per 1 mL homogenization buffer. We recommend using [^13^C_6_]IAA (Cambridge Isotope Laboratories, CLM-1896) in experiments where other [^13^C_6_]-labeled precursors (such as [^13^C_6_]anthranilate) are not used.
5. For reverse isotope dilution quantitation, use unlabeled internal standards as endogenous compounds are [^15^ N]-labeled in plants germinated on [^15^ N]ATS media. Add 50 nM ANT, 500 nM indole, 5 μM Trp, 1 nM IAM, 2.5 μM IAN, 100 nM IPyA, 10 nM IAAld, 10 nM IAA, 1 nM TAM, 10 nM IAOx and 100 mM freshly prepared methoxylamine hydrochloride (CH_3_ONH_2_ ∙ HCl) into homogenization buffer. In the data analysis output for reverse isotope dilution samples, the slope of each linear regression line represents the ratio of the respective isotopic trace (labeled compounds) to its monoisotopomer (unlabeled internal standard added) and is used to quantify the isotopic traces.
6. Although MS parameters are altered in subsequent injections, it is important to keep the LC gradient consistent with method A so that retention times are consistent across injections.
7. For absolute quantitation of compounds by reverse isotope dilution, use fully [^15^ N]-labeled salts in both germination and inhibitor media. If only relative label incorporation data is needed, unlabeled media can be used.
8. Timing for inhibitor treatments, isotopic labeling, and sample collection can be adapted to study auxin biosynthesis at different developmental stages. Significant IAA biosynthesis inhibition can be observed in 12-day seedlings (grown under 10/14-h photoperiod, cool white fluorescent lights at ~ 100 μmol m^−2^ s^−1^ at 22 °C) after 20 h on 100 μM YDF and 30 μM PVM2153 media with 30 min labeling treatment. Under these conditions, we observed greater label incorporation into IAA from labeled indole compared to other precursors. Endogenous levels of IAA precursors such as Trp may increase in the presence of biosynthesis inhibitors [20].
9. For absolute quantitation, record fresh weight of harvested tissues. This data is later used for isotope dilution calculations.
10. Recommended amounts for tissue collection: 40–50 mg per sample for [^13^C_3_]serine labeling or double indole labeling; 10–30 mg for indole/anthranilate labeling experiments. More tissue may be needed depending on plant tissue type and inhibitor treatment
11. Plant tissue should be completely pulverized after homogenization. If significant plant material remains intact, repeat homogenization step.
12. We recommend using a mass range window of the calculated *m/z* value ± 0.003.

**Table 4:** Common problems and troubleshooting guide. Adapted from Liu et al. [26]

Problem	Possible reasons	Solutions
Liquid does not pass through TopTips before loading plant samples	The slit on TopTips is too narrow	Increase the centrifugal force to make liquid pass through, or switch to a new TopTip
Liquid does not pass through TopTips after loading plant samples	Plant debris blocks the TopTip slit	Always try to avoid transferring plant debris into TopTips Increase the centrifugal force to make liquid pass through Use a dissecting probe to remove visible plant debris
Drift of LC retention time	Injection volume is too large	Evaporate eluate or extract to a smaller volume with vacuum concentrator Reduce the sample volume injected onto the LC
	Change of LC mobile phase	Check that correct, freshly prepared mobile phase solvents are being used Purge the LC lines after changing solvents
	LC solvents are blocked or leaking	Replace the precolumn filter Check column connections and reinstall if needed Check LC lines for leaking solvent
Low yield of labeled compounds, but normal yield of endogenous compounds and/or internal standards	Slow turnover	Increase the labeling time period
	Insufficient intake of labeled precursors	Increase the concentration of labeled precursors in the labeling solution Make sure plants are bathed in sufficient volume of labeling solution
Broad/tailed LC peaks	Injection volume is too large	Evaporate eluate or extract to a lower volume with vacuum concentrator Reduce the sample volume injected onto the LC
	The precolumn filter is dirty	Replace the filter
	The column is dirty	Disconnect the LC line from the MS source and wash the column with solvent according to manufacturer’s instructions
Analyte *m/z* values do not match calculated values	The MS needs to be calibrated	Perform mass calibration with calibration standard mixture according to instrument manual
Reduced MS sensitivity	The MS needs to be calibrated	See above
	The tune file needs to be modified	Adjust source parameters for optimal ion intensity using standard compounds

## Supplementary Information

**Additional file 1:** [^15^N_1_]Indole, [^2^H_5_]tryptophan, and [^13^C_6_]anthranilate labeling of IAA precursors in Arabidopsis hypocotyls in the presence of YDF.

**Additional Figure 2:** Extracted Ion Chromatogram (EIC) of targeted metabolites.

## Data Availability

The data generated in the present study are available from the corresponding author upon reasonable request.
