# Supplementary material for: Protocol: analytical methods for visualizing the indolic precursor network leading to auxin biosynthesis
Source: Plant Methods. 2021 Jun 22;17:63. doi: 10.1186/s13007-021-00763-0 (PMC8220744; doi:10.1186/s13007-021-00763-0)
Supplement: Supplementary file 1 — Additional file 1: [15N1]Indole, [2H5]tryptophan, and [13C6]anthranilate labeling of IAA precursors in Arabidopsis hypocotyls in the presence of YDF. [file 13007_2021_763_MOESM1_ESM.pptx]

## Slide 1
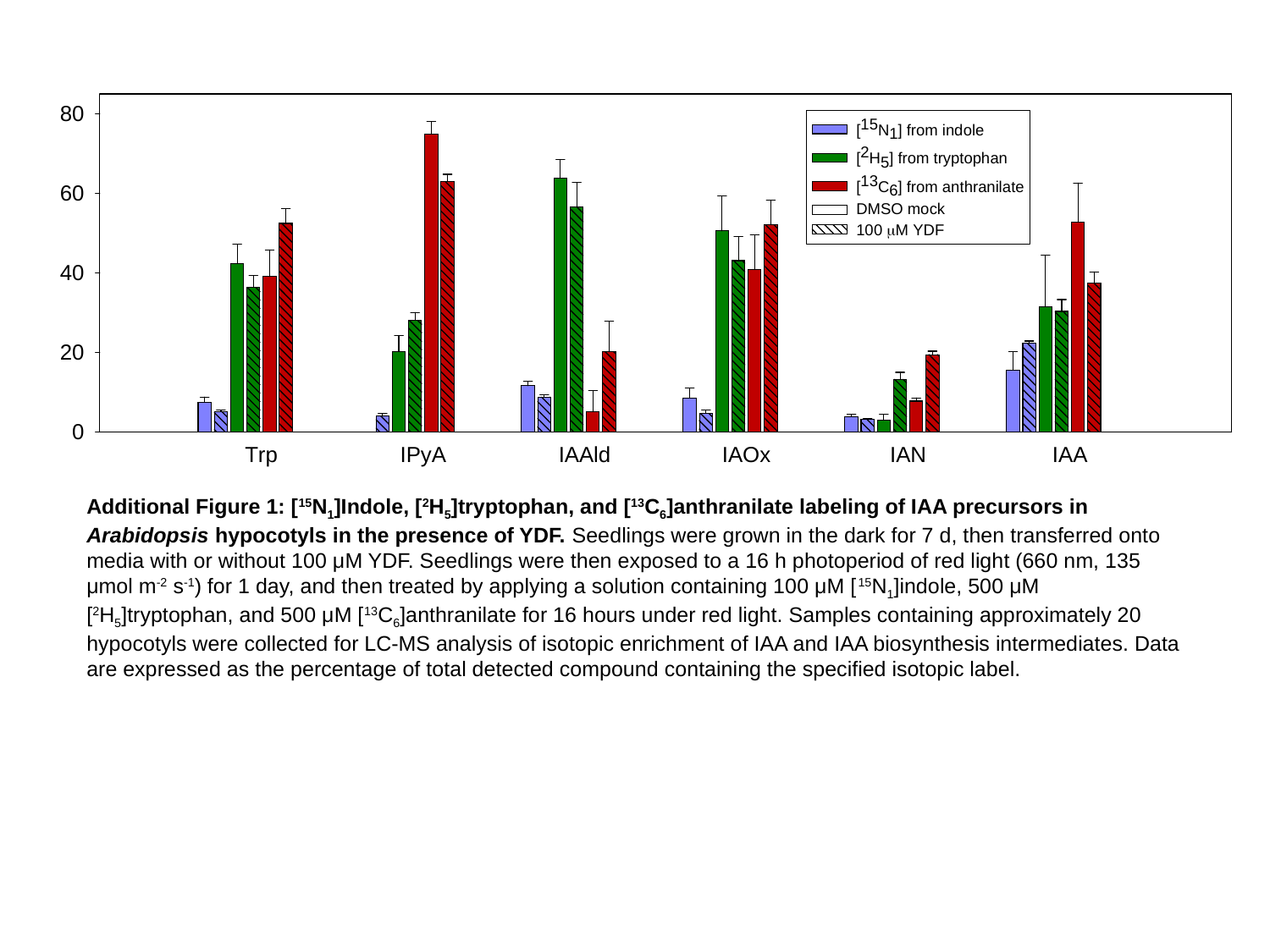

Additional Figure 1: [15N1]Indole, [2H5]tryptophan, and [13C6]anthranilate labeling of IAA precursors in Arabidopsis hypocotyls in the presence of YDF. Seedlings were grown in the dark for 7 d, then transferred onto media with or without 100 μM YDF. Seedlings were then exposed to a 16 h photoperiod of red light (660 nm, 135 μmol m-2 s-1) for 1 day, and then treated by applying a solution containing 100 μM [15N1]indole, 500 μM [2H5]tryptophan, and 500 μM [13C6]anthranilate for 16 hours under red light. Samples containing approximately 20 hypocotyls were collected for LC-MS analysis of isotopic enrichment of IAA and IAA biosynthesis intermediates. Data are expressed as the percentage of total detected compound containing the specified isotopic label.
