# Supplementary material for: Protocol: analytical methods for visualizing the indolic precursor network leading to auxin biosynthesis
Source: Plant Methods. 2021 Jun 22;17:63. doi: 10.1186/s13007-021-00763-0 (PMC8220744; doi:10.1186/s13007-021-00763-0)
Supplement: Supplementary file 2 — Additional Figure 2: Extracted Ion Chromatogram (EIC) of targeted metabolites. [file 13007_2021_763_MOESM2_ESM.pptx]

## Slide 1
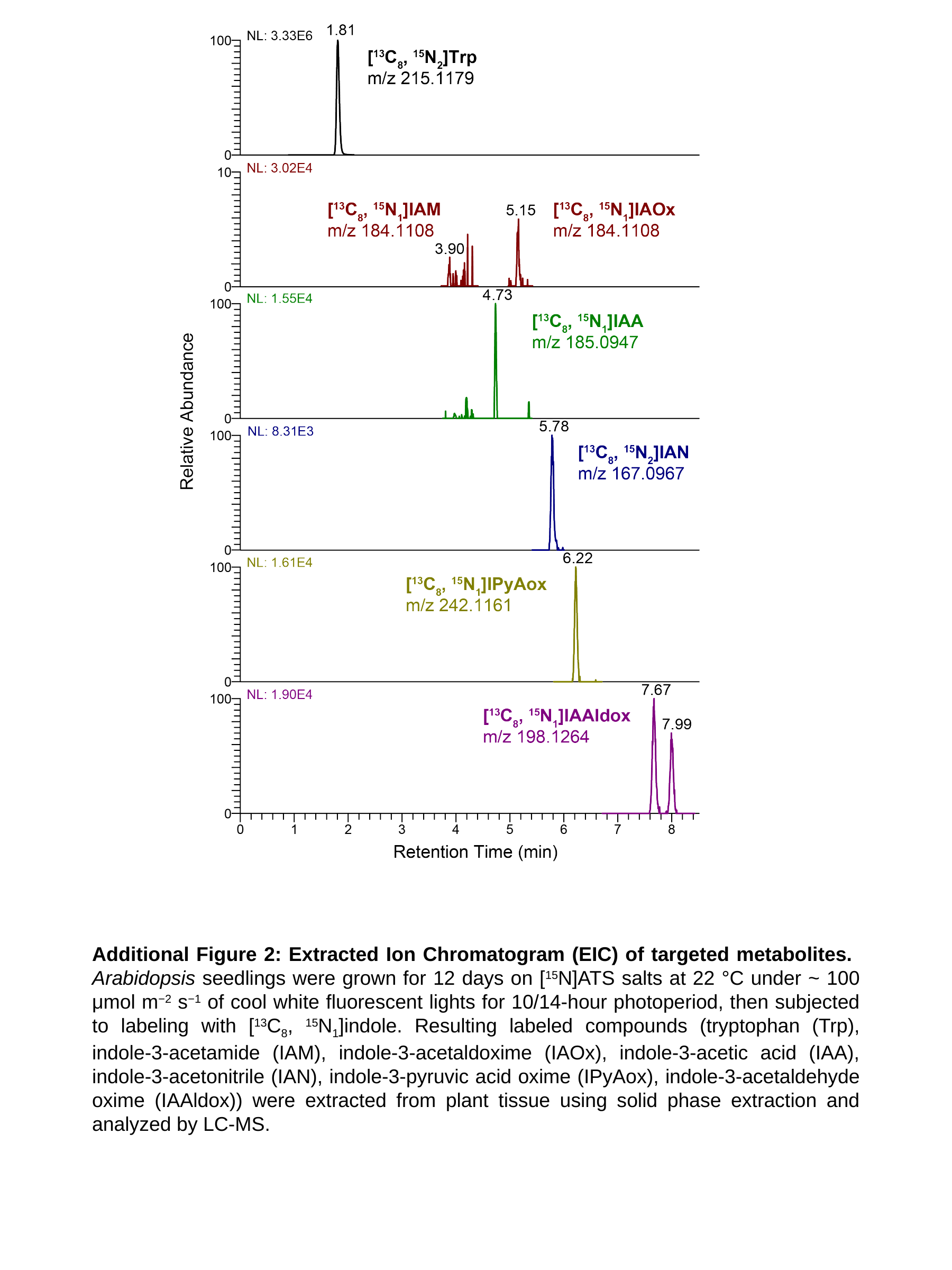

Additional Figure 2: Extracted Ion Chromatogram (EIC) of targeted metabolites. Arabidopsis seedlings were grown for 12 days on [15N]ATS salts at 22 °C under ~ 100 μmol m−2 s−1 of cool white fluorescent lights for 10/14-hour photoperiod, then subjected to labeling with [13C8, 15N1]indole. Resulting labeled compounds (tryptophan (Trp), indole-3-acetamide (IAM), indole-3-acetaldoxime (IAOx), indole-3-acetic acid (IAA), indole-3-acetonitrile (IAN), indole-3-pyruvic acid oxime (IPyAox), indole-3-acetaldehyde oxime (IAAldox)) were extracted from plant tissue using solid phase extraction and analyzed by LC-MS.
